# Supplementary material for: Systematic Identification of the RNA-Binding Protein STAU2 as a Key Regulator of Pancreatic Adenocarcinoma
Source: Cancers (Basel). 2022 Jul 26;14(15):3629. doi: 10.3390/cancers14153629 (PMC9367319; doi:10.3390/cancers14153629)
Supplement: Supplementary file 1 [file cancers-14-03629-s001.zip › cancers-1781711-supplementary.pdf]

Supplementary Materials: Systematic Identification of the RNA-Binding Protein STAU2 as a Key Regulator of Pancreatic Adenocarcinoma

Xiao Wang, Wenbin Kuang, Jiayu Ding, Jiaxing Li, Minghui Ji, Weijiao Chen, Hao Shen, Zhongrui Shi, Dawei Wang, Liping Wang and Peng Yang

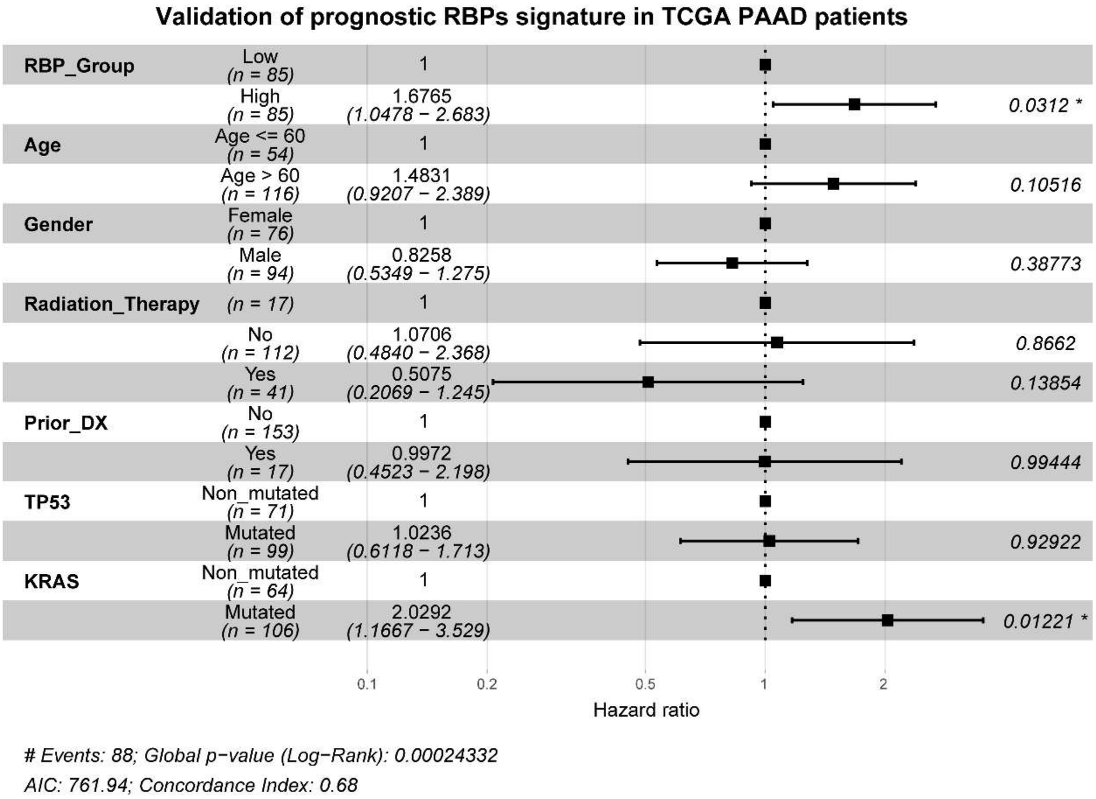

Figure S1. Validation of prognostic RBPs signature in TGCA PAAD patients. Forest plots showing the prognostic values of RBPs signature in multivariate cox regression analysis in TCGA dataset. **p* < 0.05.

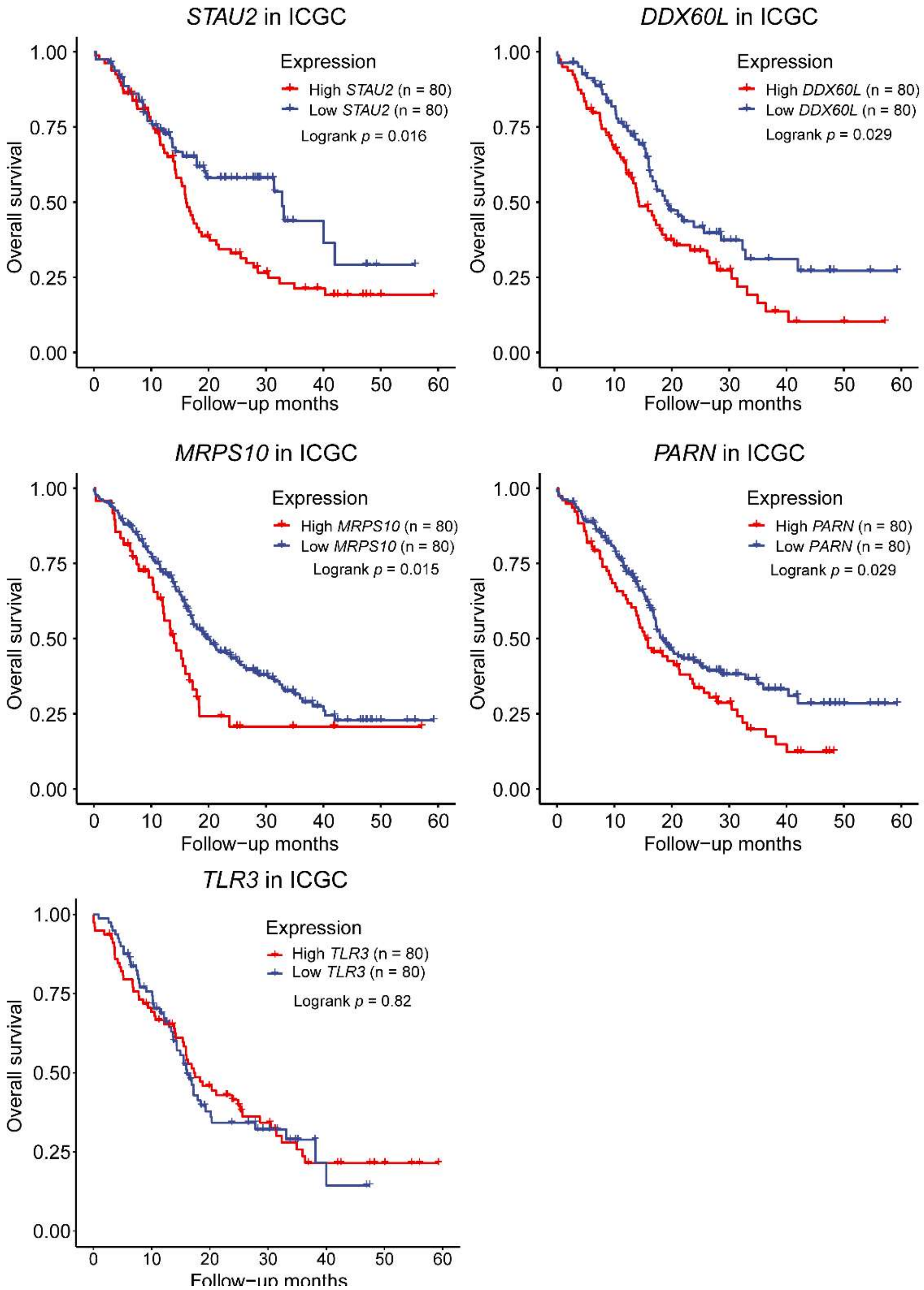

Figure S2. Validation of prognostic RBPs-hub signature in ICGC PAAD patients. Kaplan-Meier plot showing the relationship between mRNA expression of RBPs-hub signature and overall survival of ICGC PAAD patients.

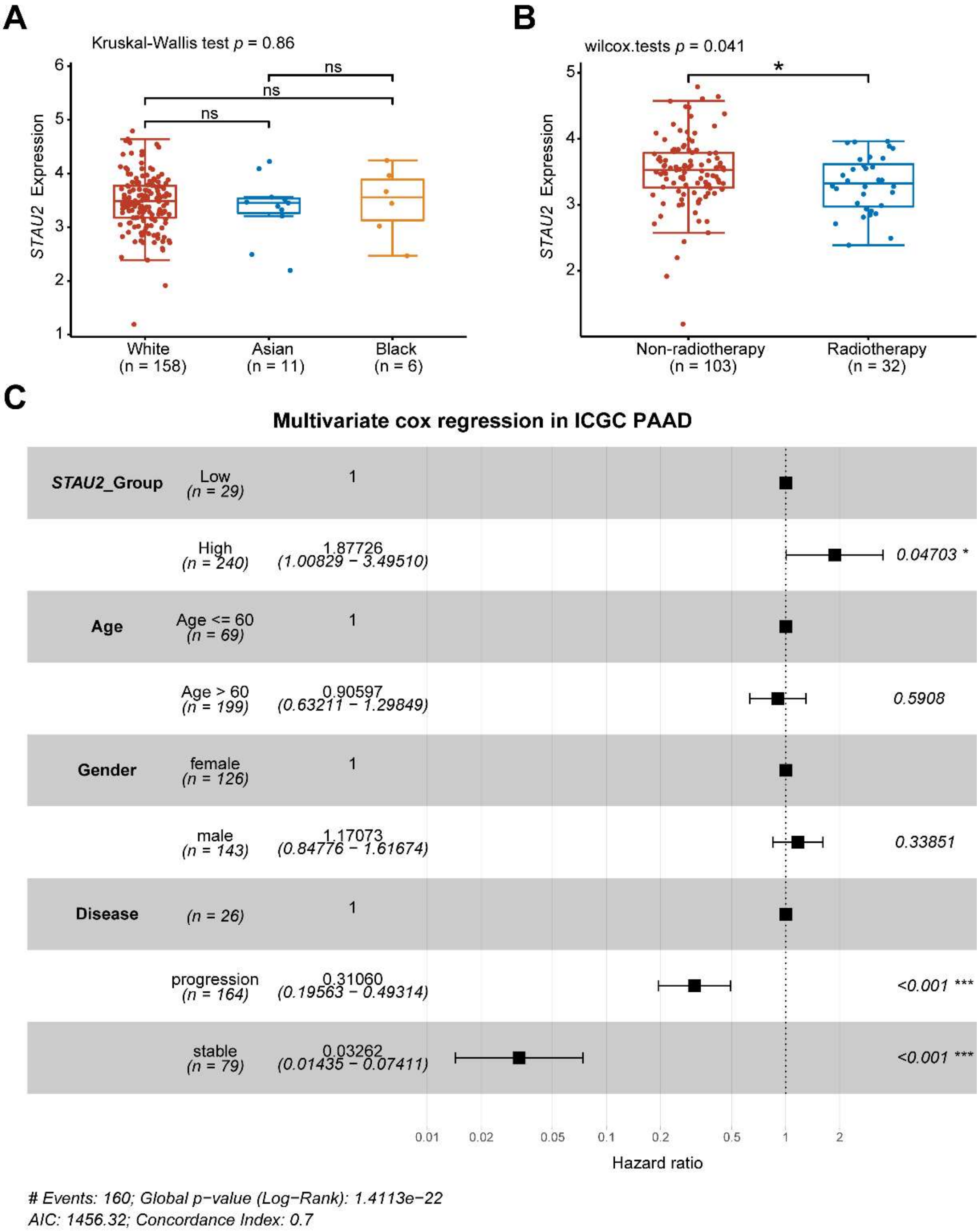

Figure S3. Geographic and clinical features specificity of STAU2 and validation of prognostic STAU2 in ICGC PAAD patients. (**A**) Differential expression of STAU2 in white, Asian and black pancreatic cancer patients. ns, $p > 0.05$. (**B**) Expression difference of STAU2 between pancreatic cancer patients with and without radiation therapy $*p < 0.05$. (**C**) Forest plots showing the prognostic values of STAU2 signature in multivariate cox regression analysis in ICGC dataset. $*p < 0.05$, $***p < 0.001$.

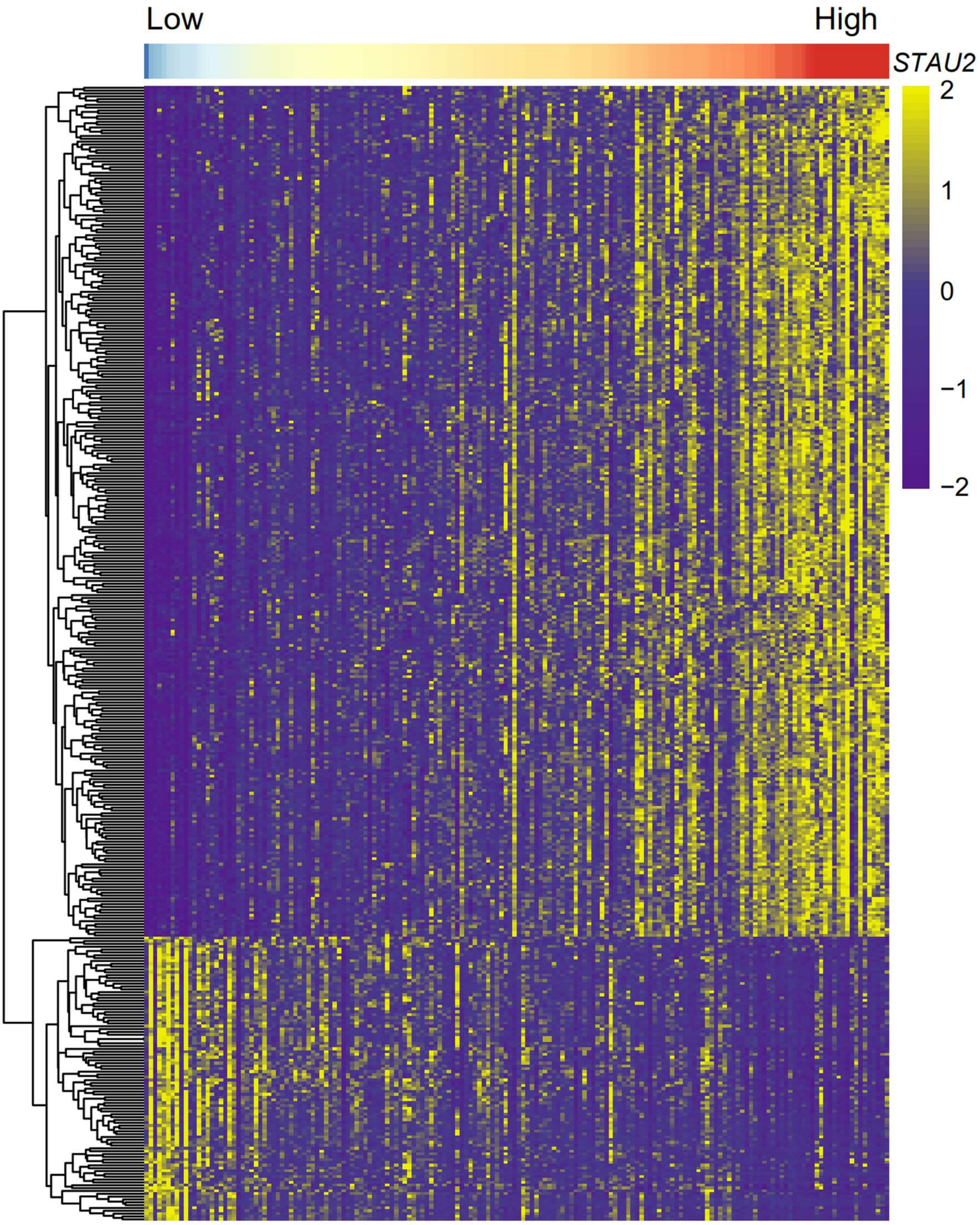

Figure S4. Heatmaps to show the expression patterns of *STAU2*-correlated genes.

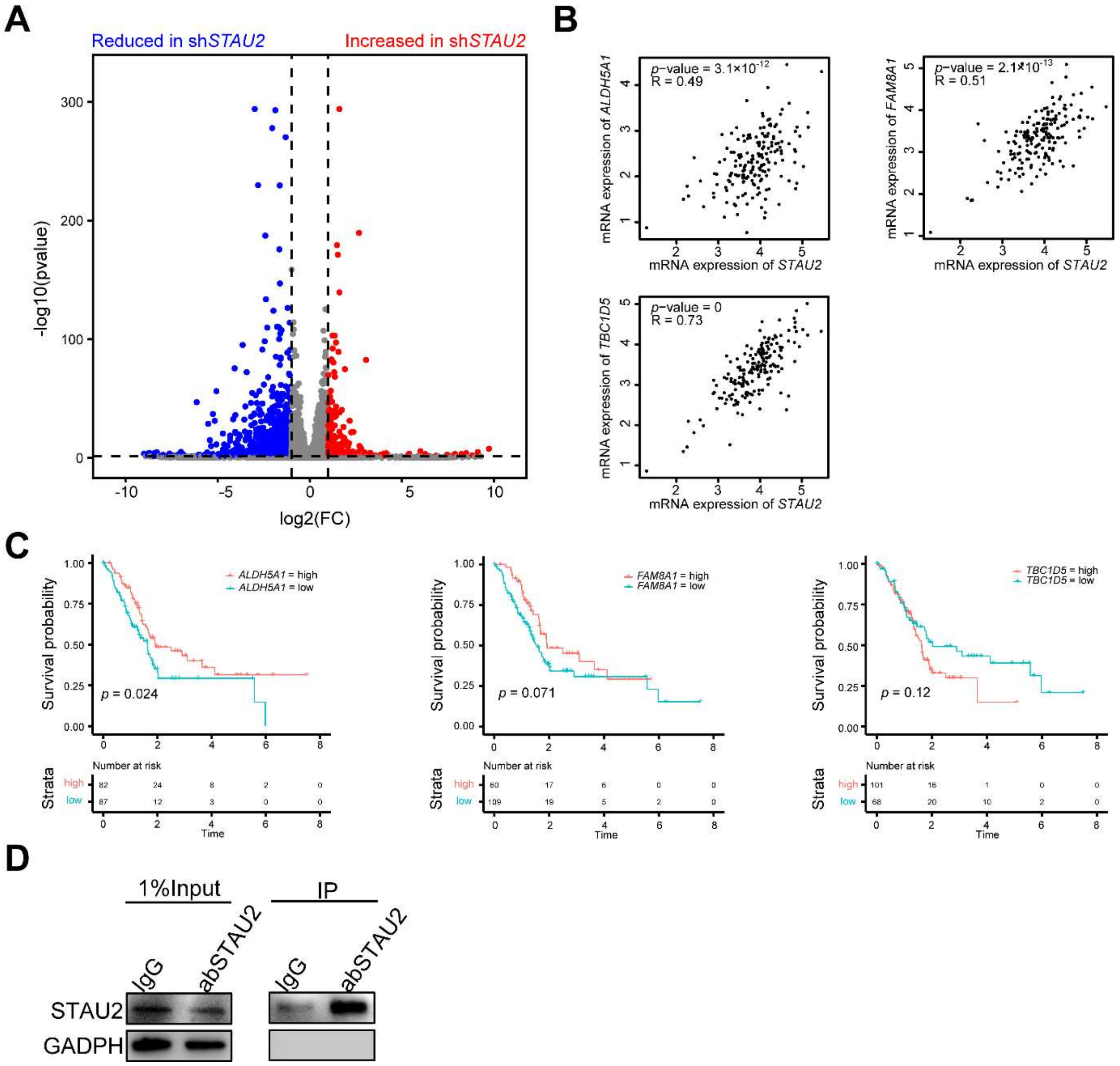

Figure S5. Validation and analysis of *STAU2* associated genes. (**A**) Genes expression of RNA sequence in sh*NC* and sh*STAU2* PANC-1 cells. (**B**) Scatter plots to show the correlation between *STAU2* and STAU2-target genes. (**C**) Kaplan-Meier plot showing the relationship between expression levels of STAU2-target genes and overall survival of TCGA-PAAD patients. (**D**) IP analysis of protein extracts from PANC-1 cells. IP was performed with a STAU2 antibody, followed by WB analysis of STAU2 pulldown products using STAU2 and GAPDH antibodies. The uncropped Western Blot images can be found in Figure S10.

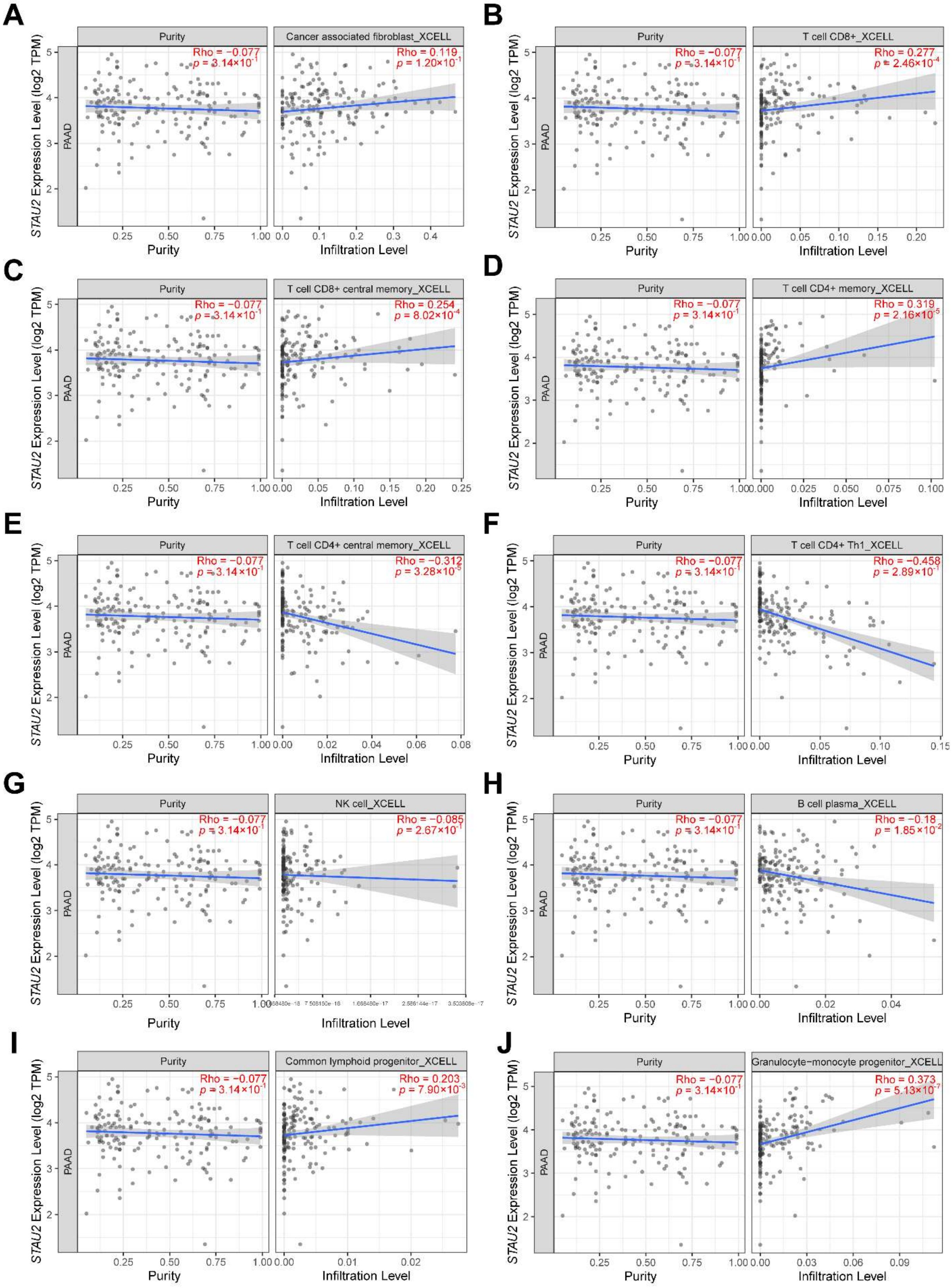

Figure S6. The association between *STAU2* expression and immune infiltration in PAAD patients. The relationship between mRNA expression levels of *STAU2* and immune infiltration levels of cancer associated fibroblast, CD8+ T-cells, CD8+ central memory T-cells, CD4+ memory T-cells, memory CD4+ central memory T-cells, Th1 CD4+ T-cells, NK T-cell, plasma B-cells, common lymphoid progenitor, granulocyte−monocyte progenitors presented by scatter plots.

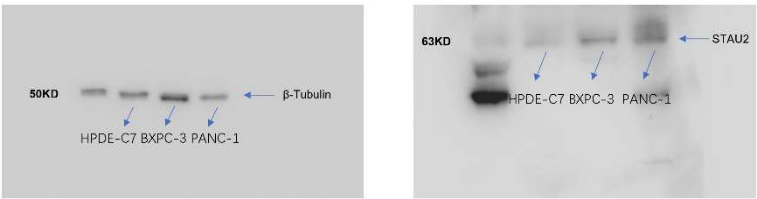

Figure S7. Uncropped Western Blot images for Figure 4F.

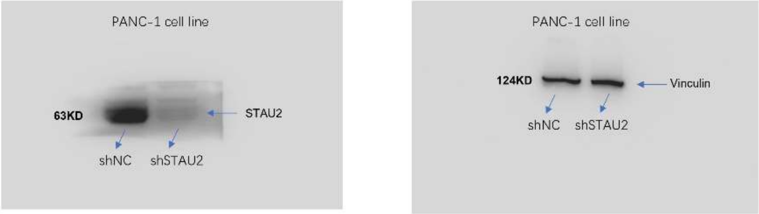

Figure S8. Uncropped Western Blot images for Figure 5A.

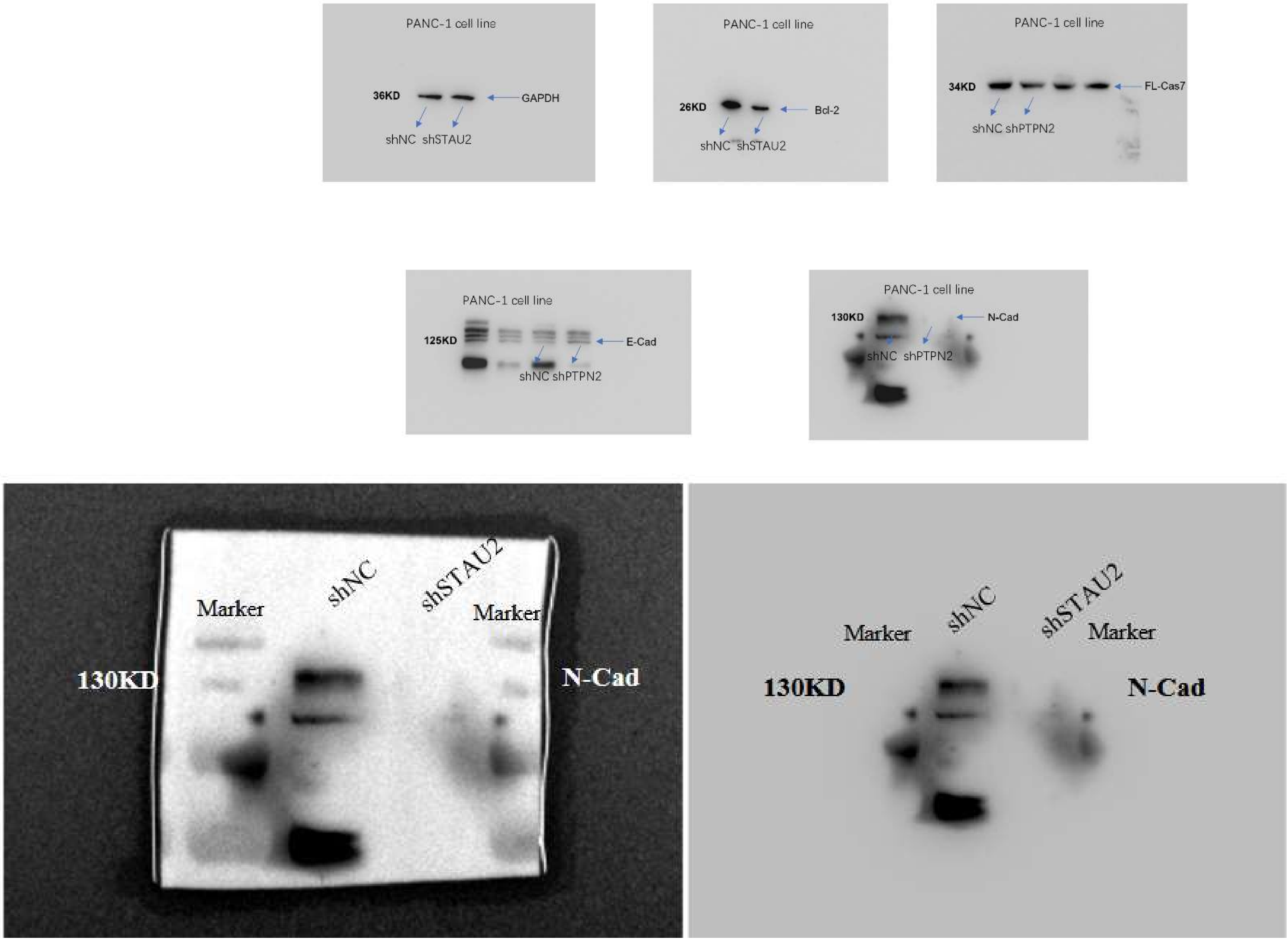

Figure S9. Uncropped Western Blot images for Figure 5F.

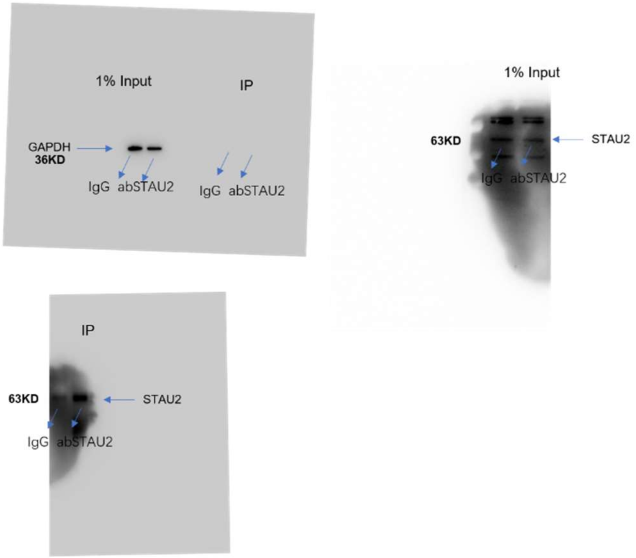

Figure S10. Uncropped Western Blot images for Figure S5D.

Table S1. Primer sequences for genes in RT-qPCR.

Name	Sense (5′–3′)	Antisense (5′–3′)
STAU2	GCTCTGAAGCGAAATATGCCTGTC	TTTAAGCTCCTGTAAGACGGTGGTCG
PALLD	GCCTACTTTCCTCCTGTTTTT	AGTGGTCATTGTTGGATTCTC
HNRNPU	GGCGGGAGGTAAGAAGAAGG	CAGGTGGCTGAGGAGATTTG
SERBP1	GTGACTGAGGAAACACCTGAA	AGCCTTCCACTCATCCAAAG
DDX3X	TGGAAATAGTCGCTGGTGTG	GGAGGACAGTTGTTGCCTGT
β-actin	CACCATTGGCAATGAGCGGTTC	AGGTCTTTGCGGATGTCCACGT

Table S2. Antibody information in Western Blot.

Name	Source	Dilution	Manufacturer	Catalong Number
β-tubulin	Rabbit	1:5000	Share-bio	AB0039
STAU2	Rabbit	1:1000	proteintech	15998-1-AP
Vinculin	Rabbit	1:1000	proteintech	26520-1-AP
GAPDH	Mouse	1:5000	proteintech	60004-1-lg
BCL2	Rabbit	1:1000	proteintech	12789-1-AP
Caspase7	Rabbit	1:1000	proteintech	27155-1-AP
E-cadherin	Rabbit	1:1000	proteintech	20874-1-AP
N-cadherin	Rabbit	1:1000	proteintech	22018-1-AP
